# Zinc ion-releasing immunomodulatory hydrogels for enhanced wound healing

**DOI:** 10.1093/rb/rbag147

**Published:** 2026-07-13

**Authors:** Yeonjeong Kim, Minjae Heo, Jeong Min Kim, Ki Dong Park, Kyung Min Park

**Affiliations:** Department of Bioengineering and Nano-Bioengineering, Incheon National University, Incheon 22012, Republic of Korea; Department of Bioengineering and Nano-Bioengineering, Incheon National University, Incheon 22012, Republic of Korea; Department of Bioengineering and Nano-Bioengineering, Incheon National University, Incheon 22012, Republic of Korea; Department of Molecular Sciences and Technology, Ajou University, Gyeonggi-do 16499, Republic of Korea; Department of Bioengineering and Nano-Bioengineering, Incheon National University, Incheon 22012, Republic of Korea; Research Center for Bio Materials & Process Development, Incheon National University, Incheon 22012, Republic of Korea

**Keywords:** polymeric hydrogels, zinc ion, immunomodulation, wound healing

## Abstract

Effective wound healing requires a pro-regenerative immune microenvironment that supports tissue regeneration. Therefore, immunomodulatory biomaterials have gained increasing attention for wound healing. In this context, hydrogels have been widely investigated owing to their biocompatibility and structural similarity to the natural extracellular matrix. However, traditional hydrogels mainly provide physical protection and moisture retention while offering limited capacity for active immune modulation. To overcome this limitation, we fabricated zinc ion (Zn^2+^)-releasing immunomodulatory hydrogels using thiol-ene chemistry, zinc peroxide (ZnO_2_)-mediated disulfide bond formation, and metal coordination. During gelation, ZnO_2_ decomposed to release therapeutic Zn^2+^. These ions were retained within the network *via* thiol-Zn^2+^ coordination, allowing sustained release for up to 28 days. Sustained Zn^2+^ release corresponded with an increased M2 macrophage population and enhanced proliferative and angiogenic responses *in vivo*. In a mouse wound model, the Zn^2+^-releasing hydrogels enhanced wound closure by creating a pro-regenerative environment. Histological analysis showed complete re-epithelialization and collagen maturation in the Zn^2+^-releasing hydrogel-treated groups. The hydrogels also increased the expression of CCL2/MCP-1, A20/TNFAIP3, and IL-13, which are involved in macrophage recruitment and transition. Overall, these results indicate that Zn^2+^-releasing hydrogels offer a promising therapy for skin regeneration by actively modulating the wound immune environment.

## Graphical Abstract

**Graphical Abstract:**
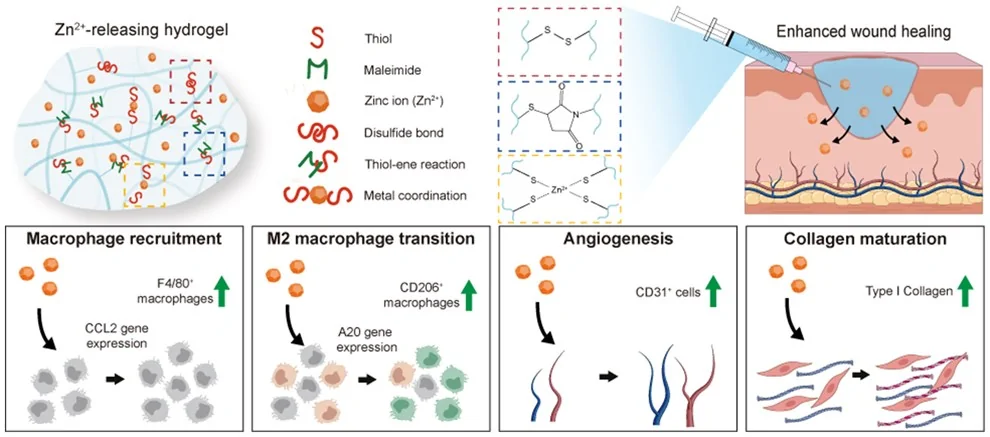

## Introduction

As the body’s largest organ, the skin serves as the first protective barrier against diverse external stimuli. Nevertheless, it is often subjected to damage from physical, chemical, and biological factors, leading to both acute and chronic wounds [1, 2]. Given that approximately 1 billion people worldwide experience various skin injuries, there remains an urgent need for regenerative therapies that restore both structural integrity and physiological function [3, 4]. The successful development of regenerative therapies relies on an understanding of the coordinated progression of wound-healing phases, as the seamless progression through each stage is essential for effective repair. Although wound healing proceeds through four phases, dysregulated inflammation can hinder the wound-healing process and compromise regeneration [5]. Accordingly, active modulation of the wound immune microenvironment has become an essential strategy for effective tissue regeneration, as reflected in recent advances in biomaterial-based approaches, including bioactive peptides [6–12], nanoparticles [13–15], and hydrogel systems [16–18].

Among various biomaterials, hydrogels are utilized for wound healing owing to their aqueous environments, biocompatibility, and similarity to the extracellular matrix (ECM) [19, 20]. While hydrogels provide physical protection and moisture retention, they are often biologically passive and offer limited capacity to regulate inflammatory signals at the wound site [21, 22]. This has stimulated increasing interest in immunomodulatory hydrogels aimed at fostering a pro-regenerative microenvironment. Specifically, macrophages serve as pivotal regulators of tissue repair, and their phenotypic transition is essential for progression through the proliferative and remodeling stages [23, 24]. Therefore, designing hydrogels that direct macrophage polarization constitutes a promising strategy to improve tissue regeneration.

In this context, inorganic ions such as zinc ion (Zn^2+^) have received considerable attention [25]. Zn^2+^ is an essential trace element that performs crucial functions in wound healing, such as immunomodulation, cell proliferation, angiogenesis, and matrix remodeling [26]. Notably, Zn^2+^ has been reported to promote a pro-regenerative immune microenvironment, thereby suppressing excessive inflammation and supporting tissue repair [22, 27]. However, previously reported Zn^2+^ delivery systems, including metal-organic frameworks and zinc nanoparticle-based hydrogels, still face several limitations, such as burst release behavior and formulation complexity [28–32]. In this regard, chemical interactions, such as thiol-Zn^2+^ coordination bonds, can help retain Zn^2+^ within hydrogel matrices and prolong its local availability. This may also contribute to the modulation of the wound immune microenvironment and tissue regeneration [33, 34].

In this study, we developed immunomodulatory Zn^2+^-releasing hydrogels aimed at promoting endogenous skin tissue regeneration. The hydrogels were synthesized through the mixing of functionalized gelatin polymers with zinc peroxide (ZnO_2_) solutions. These hydrogels exhibited prolonged Zn^2+^ release profiles, sustained for up to 28 days. The prolonged release behavior was mainly attributed to thiol-Zn^2+^ coordination inside the hydrogel network, which enabled progressive Zn^2+^ release over time. Furthermore, the Zn^2+^-releasing hydrogels promoted immunomodulation, cell proliferation, neovascularization, and collagen maturation. Notably, the hydrogels increased the expression of C-C motif chemokine ligand 2 (CCL2)/monocyte chemoattractant protein-1 (MCP-1), A20/tumor necrosis factor alpha-induced protein 3 (TNFAIP3), and interleukin 13 (IL-13) [35–39], which are involved in macrophage recruitment and polarization [40–44]. Overall, these results indicate that the Zn^2+^-releasing hydrogels are promising biomaterials for skin regeneration.

## Materials and methods

### Materials

Gelatin (type A from porcine skin, <300 bloom), cystamine dihydrochloride, 6-maleimidohexanoic acid (MHA), N-hydroxysuccinimide (NHS), N-(3-dimethylaminopropyl)-N′-ethylcarbodiimide hydrochloride (EDC), DL-dithiothreitol (DTT), Ellman’s reagent, L-cysteine, and deuterium oxide (D_2_O) were supplied by Sigma-Aldrich (St. Louis, MO, USA). Dulbecco’s Phosphate Buffered Saline (DPBS) and Superfrost Plus adhesive slides were obtained from Thermo Fisher Scientific (Rockford, IL, USA). 1 N Hydrochloric acid (HCl), Tris-HCl (1 M, pH 8.0), dialysis membranes (MWCO 3.5 kDa), and ZnO_2_ were obtained from Daejung (Siheung, Gyeonggi, South Korea), Invitrogen (Grand Island, NY, USA), Spectrum Laboratories (Rancho Dominguez, CA, USA), and Alfa Aesar (Haverhill, MA, USA), respectively [45, 46].

Human dermal fibroblasts (HDFs) were obtained from Lonza (Walkersville, MD, USA). Dulbecco’s Modified Eagle’s Medium with high glucose (DMEM-HG), fetal bovine serum (FBS), and penicillin/streptomycin (P/S) were supplied by Gibco (Grand Island, NY, USA). The zinc assay kit, WST-1 reagent, and live/dead kit were obtained from Sigma-Aldrich, Roche (Basel, Switzerland), and Invitrogen, respectively.

For histological and immunofluorescence (IF) analyses, Histoplast IM, Harris hematoxylin, eosin Y, and 10% formalin were obtained from Sigma-Aldrich. Anti-CD206, anti-Ki67, anti-CD31 antibodies, goat anti-rabbit IgG H&L (Alexa Fluor 568), and donkey anti-rabbit IgG H&L (Alexa Fluor 488) were obtained from Abcam (Cambridge, UK). Anti-F4/80 antibody and F4/80 monoclonal antibody (BM8) were obtained from Genetex (Irvine, CA, USA) and Thermo Fisher Scientific, respectively. 4ʹ,6-diamidino-2-phenylindole (DAPI), Tegaderm™, and isoflurane were purchased from Probes (Eugene, OR, USA), 3M Health Care (St. Paul, MN, USA), and Choongwae Pharm (Seoul, South Korea), respectively. Quantitative reverse transcription polymerase chain reaction (qRT-PCR) primers and Herovici’s staining kit were supplied by Cosmogenetech (Seongdong-gu, Seoul, South Korea) and StatLab (McKinney, TX, USA), respectively.

### Animal studies

All animal experiments were approved by the Institutional Animal Care and Use Committee of Incheon National University (INU-ANIM-2022-14). Female C57BL/6N mice (5 weeks old; Koatech, Pyeongtaek, South Korea) were acclimated for 1 week before use. Animals were housed under specific pathogen-free conditions at 22 ± 2°C on a 12 h light/dark cycle with free access to food and water.

### Synthesis and characterization of functionalized polymers

Thiolated gelatin (GtnSH) (×1/2 and ×1) was prepared by introducing cystamine into gelatin, followed by reductive cleavage of the disulfide moiety. Both polymers were prepared by modifying a previously reported protocol [47], with changes in the cystamine feed ratio and reduction conditions as described below. Gelatin (250 mg) was dissolved in deionized water (DIW) (125 mL, 35–40°C), and cystamine dihydrochloride was added at 1.0 mmol for GtnSH (×1/2) or 2.0 mmol for GtnSH (×1). The EDC/NHS feed levels and DTT reduction amounts were adjusted accordingly: 0.5:0.5 mmol EDC/NHS and 2.0 mmol DTT for GtnSH (×1/2), and 1.0:1.0 mmol EDC/NHS and 4.0 mmol DTT for GtnSH (×1). The mixture reacted for 2 h, followed by DTT reduction for an additional 24 h.

Maleimide-conjugated gelatin (GtnMI) was synthesized through carbodiimide coupling of MHA to the gelatin backbone as described in previous studies [48]. Gelatin (250 mg) was dissolved in DPBS (50 mL, 35–40°C), and a separately prepared MHA solution in DPBS was added under light-protected conditions (35–40°C). EDC and NHS were then added to the reaction mixture at an MHA:EDC:NHS molar ratio of 1:1.2:1.4, and the reaction proceeded for 2 h. After dialysis against DIW for 72 h (MWCO: 3.5 kDa), the purified polymer was freeze-dried and stored at −80°C in the dark until use.

The chemical structure of GtnSH was analyzed by proton nuclear magnetic resonance spectroscopy (^1^H-NMR, Agilent 400-MR, Agilent Technologies, CA, USA). Each polymer sample was dissolved in D_2_O at 10 mg/mL and analyzed [49]. The amount of free thiol groups in GtnSH was determined by Ellman’s assay, as previously reported [45, 50]. Briefly, GtnSH was dissolved in DIW at 1 mg/mL, reacted with Ellman’s reagent for 20 min, and absorbance was measured at 405 nm using a UV spectrophotometer. Free thiol content was calculated as μmol of thiol groups per gram of GtnSH from a cysteine standard curve (0.001–0.04 mg/mL).

The chemical structure of GtnMI was analyzed by ^1^H-NMR spectroscopy. Each polymer sample was dissolved in D_2_O at 10 mg/mL and analyzed [49]. The amount of maleimide present on the polymer chain was determined indirectly by measuring the decrease in free cysteine with Ellman’s reagent, as previously reported [46, 47]. Briefly, GtnMI (1 mg/mL) and cysteine (0.04 mg/mL) solutions prepared in DIW were mixed and incubated in the dark for 10 min, followed by reaction with Ellman’s reagent for 20 min. Absorbance was recorded at 405 nm, and maleimide content was determined from the decrease in free cysteine using a cysteine standard curve.

### Preparation of the Zn^2+^-releasing hydrogels and phase transition time

The Zn^2+^-releasing hydrogels were formed by simply mixing GtnSH, GtnMI and ZnO_2_ solutions immediately before use. All functionalized polymers were dissolved in DPBS for hydrogel fabrication. Phase transition time was measured as a function of hydrogel composition, including polymer concentration, thiol-to-maleimide equivalence, and ZnO_2_ concentration. To examine gelation behavior, formulations were varied by total polymer content (3–7 wt%), thiol equivalence of GtnSH (×1/2 and ×1), and ZnO_2_ concentration (0–0.25 wt%). For ZnO_2_-containing formulations, 25 μL of GtnSH solution (6 wt%, ×1/2), 22.5 μL of GtnMI solution (6.67 wt%), and 2.5 μL of ZnO_2_ solution in 0.2 M Tris-HCl were mixed. Final group compositions are summarized in Table 1. Phase transition time was determined by the vial-inversion method and defined as the time point at which macroscopic flow was no longer observed [45, 51].

**Table 1:** Group codes of hydrogels.

Group name	GtnSH (×1/2) (wt%)	GtnMI (wt%)	ZnO_2_ (wt%)	Tris-HCl (M)
Z0	3	3	0	0.01
Z0.01			0.01	
Z0.05			0.05	
Z0.25			0.25	

### Rheological analysis of the Zn^2+^-releasing hydrogels

Rheological measurements were performed to characterize the mechanical properties of the hydrogels using a DHR-1 rheometer (TA Instruments, New Castle, USA). Zn^2+^-releasing hydrogels (200 μL) were loaded onto the instrument and analyzed using a 20-mm parallel-plate geometry at a gap of 600 μm. Dynamic time sweeps were conducted at 37°C with 0.1% strain and 0.1 Hz frequency for samples containing different ratios and concentrations of GtnSH, GtnMI, and ZnO_2_. To reduce evaporation during testing, DIW was placed in the solvent trap [45, 51–54].

### Releasing kinetics of Zn^2+^ and H_2_O_2_

Zn^2+^ release was analyzed using a zinc-ligand binding assay. Hydrogels (20 μL) containing different amounts of ZnO_2_ (0.01–0.25 wt%) were placed in 48-well plates, immersed in 500 μL of sterile DPBS, and incubated at 37°C with 5% CO_2_. The release medium was collected and replaced with fresh DPBS at set time points over 28 days. The zinc concentration in each eluate was measured using a commercial zinc assay kit, and cumulative release was expressed as the total molar amount of Zn^2+^ released from the gels.

H_2_O_2_ release was measured in parallel using the Cu(II)-neocuproine assay. Hydrogels were incubated under the same conditions described above. At each time point, the entire supernatant was removed and replaced with fresh DPBS. For analysis, 0.01 M phosphate buffer, hydrogel eluate or an H_2_O_2_ standard, 0.01 M CuSO_4_, and 0.01 M neocuproine solution were mixed and reacted for 20 min. After the reaction, absorbance at 450 nm was recorded with a UV spectrophotometer. H_2_O_2_ concentration was calculated using a standard curve ranging from 0 to 1000 μM [45, 52, 53].

Residual zinc distribution within the hydrogels was examined by SEM-EDS (JSM 7800F, JEOL, Tokyo, Japan). For sample preparation, 100 μL of hydrogel samples were fabricated in 1-mL syringes and incubated for 28 days under the same release conditions. After incubation, the samples were sectioned, lyophilized, sputter-coated with gold, and subjected to SEM-EDS analysis [52].

### Surface chemical state analysis

Surface analysis of the hydrogels was performed by X-ray photoelectron spectroscopy (XPS; NEXSA G2, Thermo Fisher Scientific) using a monochromated Al Kα source. Samples were prepared in the same manner as those used for elemental analysis. After fabrication, the hydrogels were rinsed 10 times with DIW, sectioned, and freeze-dried. Survey spectra were acquired over the range of 0–1350 eV under high vacuum. Binding energies were referenced to the C 1s peak at 284.8 eV. For spectral fitting, the S 2p doublets were constrained to a spin-orbit splitting of 1.20 eV and an area ratio of 2:1, whereas the Zn 2p doublets were fitted using a fixed splitting of 23.3 eV and a 2:1 area ratio. These fitting constraints were applied using Avantage software (version 5.9931) to compare sulfur- and zinc-related chemical states in the hydrogel network [55].

### Cytocompatibility of the Zn^2+^-releasing hydrogels

Cytocompatibility was assessed using HDFs cultured in DMEM-HG supplemented with 10% FBS and 1% P/S (37°C, 5% CO_2_). Cells were seeded in 48-well plates at 1.5 × 10^4^ cells/well and allowed to attach for 24 h before treatment with 20 μL of each hydrogel formulation. After an additional 24 h, cell viability was quantified using a WST-1 assay. Cell viability was further visualized by optical microscopy (Eclipse TS100, Nikon, Tokyo, Japan) and calcein-AM/EthD-1 live/dead staining followed by fluorescence microscopy (Eclipse Ti-2, Nikon) [45].

### *In vivo* subcutaneous implantation

Subcutaneous implantation was performed to evaluate tissue compatibility and host responses to the Zn^2+^-releasing hydrogels. After acclimation, 6-week-old female C57BL/6N mice were randomly assigned to four groups comprising a Zn^2+^-free group (Z0) and three Zn^2+^-releasing groups (Z0.01, Z0.05, and Z0.25). After the mice were anesthetized with isoflurane, the dorsal skin was shaved and disinfected. Then, 200 μL of hydrogel preloaded in a 1-mL syringe was implanted subcutaneously into the dorsal region. Animals were sacrificed on day 14 for histological analysis and assessment of immunomodulatory gene expression. For tissue compatibility evaluation, an additional set of animals was sacrificed on day 35 after complete hydrogel degradation. The heart, liver, spleen, lung, and kidney were fixed in 10% formalin for 7 days before further histological analysis [53].

### *In vivo* gene expression

To analyze gene expression in cells infiltrating the hydrogel, subcutaneously implanted samples were retrieved after 14 days. The explanted hydrogels were enzymatically digested with collagenase (1 mg/mL; C6885, Sigma-Aldrich) for 3 h to isolate infiltrated cells. RNA was extracted from the recovered cell suspension using TRIzol (Invitrogen, USA) according to the manufacturer’s protocol. The extracted RNA was used for cDNA synthesis with PrimeScript RT Master Mix (TaKaRa, Japan) on a T100 thermal cycler (Bio-Rad, USA).

qRT-PCR was conducted using a StepOnePlus Real-Time PCR system (Applied Biosystems, USA). Primer sequences are listed in Table 2. Expression levels were normalized to GAPDH and presented as relative values. All experiments were independently repeated three times.

**Table 2:** Primer sequences used in the qRT-PCR analysis.

Category	Genes	Forward	Reverse
Mouse	GAPDH	5ʹ-GGT GAA GGT CGG TGT GAA CG-3ʹ [56]	5ʹ-CTC GCT CCT GGA AGA CGG TG-3ʹ
	CCL2/MCP-1	5ʹ-TAA AAA CCT GGA TCG GAA CCA AA-3ʹ	5ʹ-GCA TTA GCT TCA GAT TTA CGG GT-3ʹ
	A20/TNFAIP3	5ʹ-GAA CAG CGA TCA GGC CAG G-3ʹ	5ʹ-GGA CAG TTG GGT GTC TCA CAT T-3ʹ
	IL-13	5ʹ-CAG CCT CCC CGA TAC CAA AAT-3ʹ	5ʹ-GCG AAA CAG TTG CTT TGT GTA G-3ʹ

### *In vivo* wound closure

The wound-healing efficacy of the hydrogels was evaluated in a murine full-thickness skin defect model. Following a 1-week acclimation, the dorsal skin was shaved under isoflurane anesthesia, and a silicone splint was sutured to reduce wound contraction. An 8-mm full-thickness wound was fabricated on the mouse dorsum. Each wound was treated with 50 μL of the designated hydrogel formulation, comprising a Zn^2+^-free group (Z0) and three Zn^2+^-releasing groups (Z0.01, Z0.05, and Z0.25) and was covered with Tegaderm (3M, USA).

Following the procedure, all mice were individually housed and received intraperitoneal injections of gentamicin and ketoprofen (Sigma-Aldrich). Wounds were photographed on days 0, 3, 7, 11, and 14, and wound areas were measured using ImageJ (National Institutes of Health, MD, USA). Additionally, the wound closure (%) was determined using the following formula:


$$\text{Wound}\;\text{closure}\left(\%\right)=\left(A_{0}-A_{i}\right)/A_{0}\times 100\%,$$

where A_0_ is the wound area on day 0 and A_i_ is the wound area on a given day. Regenerated wounds were collected on day 14 for histological analysis [45].

### Histological analysis and IF staining

For histological evaluation, explanted hydrogels together with the surrounding tissue were collected after 14 days and fixed in 10% formalin. Samples were dehydrated, embedded in paraffin at 58°C, and sectioned at 4 μm. Sections were mounted on adhesive slides. H&E staining was performed to assess tissue morphology and cellular infiltration, and Herovici’s staining was used to evaluate collagen deposition in regenerated wound tissue following the manufacturer’s instructions.

For IF staining, sections were subjected to deparaffinization, antigen retrieval, and blocking with a 5 wt% BSA solution for 1 h at 4°C. Sections were treated with primary antibodies against F4/80 (1:1000), CD206 (1:1000), Ki67 (1:2000) or CD31 (1:2000) overnight at 4°C. After washing, sections were incubated with Alexa Fluor 568 (1:1000) for 1 h at 25°C.

For F4/80/CD206 double staining, sections were first incubated with F4/80 monoclonal antibody (1:500) for 1 h at 25°C and then with anti-CD206 antibody (1:1000) overnight at 25°C, followed by incubation with Alexa Fluor 488 (1:500) for 1 h. Nuclei were counterstained using DAPI (1:500) for 2 min. IF images for F4/80, Ki67, and CD31 were acquired using a fluorescence microscope, and F4/80/CD206 double-stained sections were imaged by confocal microscopy (ZEISS LSM 700, Jena, Germany). Quantification was performed using ImageJ.

### Statistical analysis

Data are presented as mean ± SD. Sample sizes (*n*), representing independent biological replicates, are indicated in the corresponding figure legends. Two-group comparisons were performed using an unpaired Student’s *t*-test. Multiple-group comparisons were analyzed by one-way analysis of variance (ANOVA) followed by Tukey’s *post hoc* test. Time-course wound closure data were analyzed by two-way ANOVA followed by Tukey’s *post hoc* test. Statistical analyses were performed using GraphPad Prism 8.0.2 (GraphPad Software, Inc., CA). Statistical significance was defined as *P < 0.05, **P < 0.01, ***P < 0.001, and ****P < 0.0001.

## Results and discussion

### Fabrication and characterization of the Zn^2+^-releasing hydrogels

We fabricated Zn^2+^-releasing immunomodulatory hydrogels by utilizing functionalized gelatin polymers and ZnO_2_. Gelatin served as the main backbone polymer due to its favorable biocompatibility, degradability, and cost-effectiveness [47, 57]. In addition, gelatin-based polymers provide intrinsic cell-adhesion motifs and diverse functional groups that facilitate cell infiltration and enable further chemical modification [58]. Furthermore, ZnO_2_ undergoes decomposition to release Zn^2+^ and H_2_O_2_, which function as therapeutic cues for wound healing and as an oxidative stimulus for crosslinking, respectively [59].

Leveraging these advantages, hydrogels were fabricated by simply mixing GtnSH, GtnMI, and ZnO_2_ solutions at a volume ratio of 10:9:1. This process initiated simultaneous crosslinking through a thiol-ene reaction, ZnO_2_-mediated disulfide bond formation, and metal coordination (Figure 1A). Within this system, the gradual decomposition of ZnO_2_ provided Zn^2+^ to the wound area.

**Figure 1:**
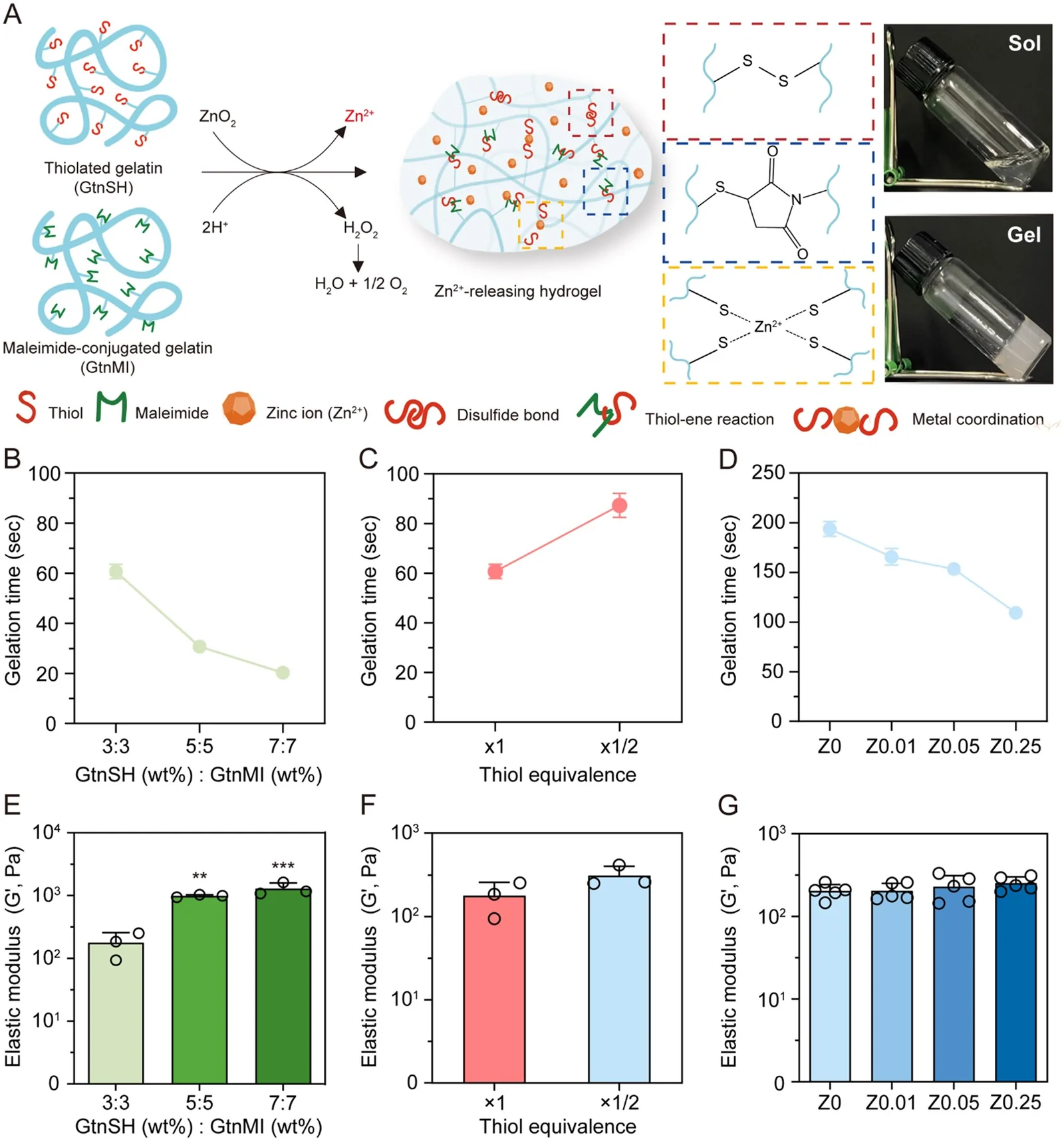
Physicochemical properties of the Zn^2+^-releasing hydrogels. (**A**) Schematic illustration of the Zn^2+^-releasing hydrogel fabrication process and digital images of the sol–gel phase transition. Phase transition time of hydrogel formulations as a function of (**B**) polymer concentration, (**C**) thiol equivalence, and (**D**) ZnO_2_ concentration. Rheological properties of hydrogels with varying (**E**) polymer concentration, (**F**) thiol equivalence, and (**G**) ZnO_2_ concentration. Data in (**E, G**) are presented as mean ± SD (*n* = 3 and *n* = 5, respectively) and were analyzed by one-way ANOVA with Tukey’s multiple comparisons test. Data in (**F**) are presented as mean ± SD (*n* = 3) and were analyzed by a two-tailed unpaired *t*-test. * indicates significant differences compared to the 3:3 group (**P < 0.01 and ***P < 0.001).

First, we investigated the sol-gel phase transition time, which influences *in situ* applicability. A controlled gelation time is essential, as excessively rapid or slow gelation can reduce hydrogel stability or application efficiency. To quantify the phase transition time, a vial-tilting assay was performed while varying the polymer concentration (3–7 wt%), thiol equivalence of GtnSH (×1/2 and ×1), and ZnO_2_ concentration (0–0.25 wt%). Increasing the total polymer content decreased the sol–gel transition time from 61 to 20 s (Figure 1B), indicating that higher concentrations of thiol and maleimide groups promoted faster network formation. In addition, the phase transition time could be tuned by adjusting the thiol equivalence. Hydrogels formulated with GtnSH (×1) crosslinked in 61 s, whereas those with GtnSH (×1/2) formed in 87 s (Figure 1C). Furthermore, increasing the ZnO_2_ concentration reduced the phase transition time from 194 to 110 s (Z0, 194 s; Z0.01, 166 s; Z0.05, 154 s; Z0.25, 110 s) (Figure 1D). These findings suggest that the increased density of functional groups and the incorporation of ZnO_2_ accelerate the thiol-ene reaction, disulfide bond formation, and metal coordination. Thus, these results demonstrate that gelation behavior is controllable by modulating polymer content and functional groups to suit specific requirements.

The mechanical characteristics of biomaterials play an essential role not only in preserving the structural stability of hydrogels but also in influencing cellular behaviors, including migration, differentiation, and proliferation [46, 60]. To evaluate these properties, we measured the storage modulus (G′) using a rheological dynamic time sweep. The G′ value increased with polymer concentration, rising from 180 to 1,300 Pa (Figure 1E). This trend was directly attributed to the enhanced thiol-ene crosslinking density. By adjusting the thiol equivalence, the mechanical strength was tuned to 180 Pa for GtnSH (×1) and 310 Pa for GtnSH (×1/2) (Figure 1F). This demonstrates that mechanical properties are maximized as the stoichiometry of thiol and maleimide groups approaches a 1:1 molar ratio (thiol content: GtnSH (×1), 168 μmol/g; GtnSH (×1/2), 86 μmol/g; maleimide content: GtnMI, 102 μmol/g). Additionally, increasing the ZnO_2_ concentration raised G′ from 200 to 250 Pa (Figure 1G). These results indicate that ZnO_2_ incorporation further reinforces the hydrogel network, thereby enhancing its structural stability for wound healing applications.

While maintaining structural stability is essential, promoting cellular infiltration into the hydrogel matrix is equally critical for tissue regeneration. To confirm the degree of cell infiltration in relation to G′ values, the hydrogels were implanted into mice (Supplementary Figure S1A). Following harvest, H&E staining was performed to assess cell recruitment depending on polymer concentration and thiol equivalence (Supplementary Figure S1B) or ZnO_2_ concentration (Supplementary Figure S1C). This analysis revealed that increasing the polymer concentration significantly reduced the number of infiltrated cells (Supplementary Figure S1D). In contrast, different thiol equivalence levels did not result in statistically significant differences in infiltrating cell numbers (Supplementary Figure S1E). In addition, cell infiltration increased in the Z0.25 group (Supplementary Figure S1F). These findings demonstrate that significant increases in hydrogel stiffness can act as a physical barrier that limits cellular influx within the implanted scaffold.

Based on these characterizations, we aimed for a sol–gel transition time exceeding 60 s to allow sufficient time for homogeneous mixing and uniform hydrogel formation. Additionally, maximizing cellular infiltration was prioritized to facilitate tissue integration. To fulfill this requirement, we selected the hydrogel consisting of 3 wt% GtnSH (×1/2) and 3 wt% GtnMI. This optimized formulation was employed for all subsequent experiments, and for clarity, GtnSH (×1/2) is hereafter referred to as GtnSH.

### Release kinetics of Zn^2+^ and H_2_O_2_

In our hydrogel system, ZnO_2_ undergoes decomposition to release Zn^2+^, generating H_2_O_2_ as an intermediate molecule. While Zn^2+^ is an essential micronutrient involved in wound healing, excessive concentrations can induce cytotoxicity [61–63]. In the same manner, H_2_O_2_ promotes disulfide bond formation under oxidative conditions. However, concentrations above 200 μM have been reported to be cytotoxic [64]. Therefore, to ensure cytocompatibility while maintaining therapeutic efficacy, we quantified released Zn^2+^ using a zinc-ligand binding assay and measured H_2_O_2_ levels by Cu(II)-neocuproine spectrophotometry.

The Zn^2+^-releasing hydrogels containing different amounts of ZnO_2_ (0–0.25 wt%) were fabricated, and eluate samples were obtained at designated time points. Released Zn^2+^ concentrations increased in proportion to ZnO_2_ content (Z0.01, 3.8 μM; Z0.05, 39.2 μM; Z0.25, 114.7 μM on day 28), with sustained release observed for up to 28 days (Figure 2A). This trend indicates that ZnO_2_ concentration influences the duration of Zn^2+^ release. Sustained Zn^2+^ release for up to 28 days was observed in the Z0.25 group, whereas the Z0.01 and Z0.05 groups reached a plateau earlier. Remarkably, the cumulative release remained below 4% of the initial zinc concentration across all groups (Z0.01, 3.8%; Z0.05, 0.8%; Z0.25, 0.5% on day 28) (Figure 2B). These findings indicate that the hydrogel matrix effectively retains Zn^2+^, preventing burst release and enabling long-term sustained delivery.

**Figure 2:**
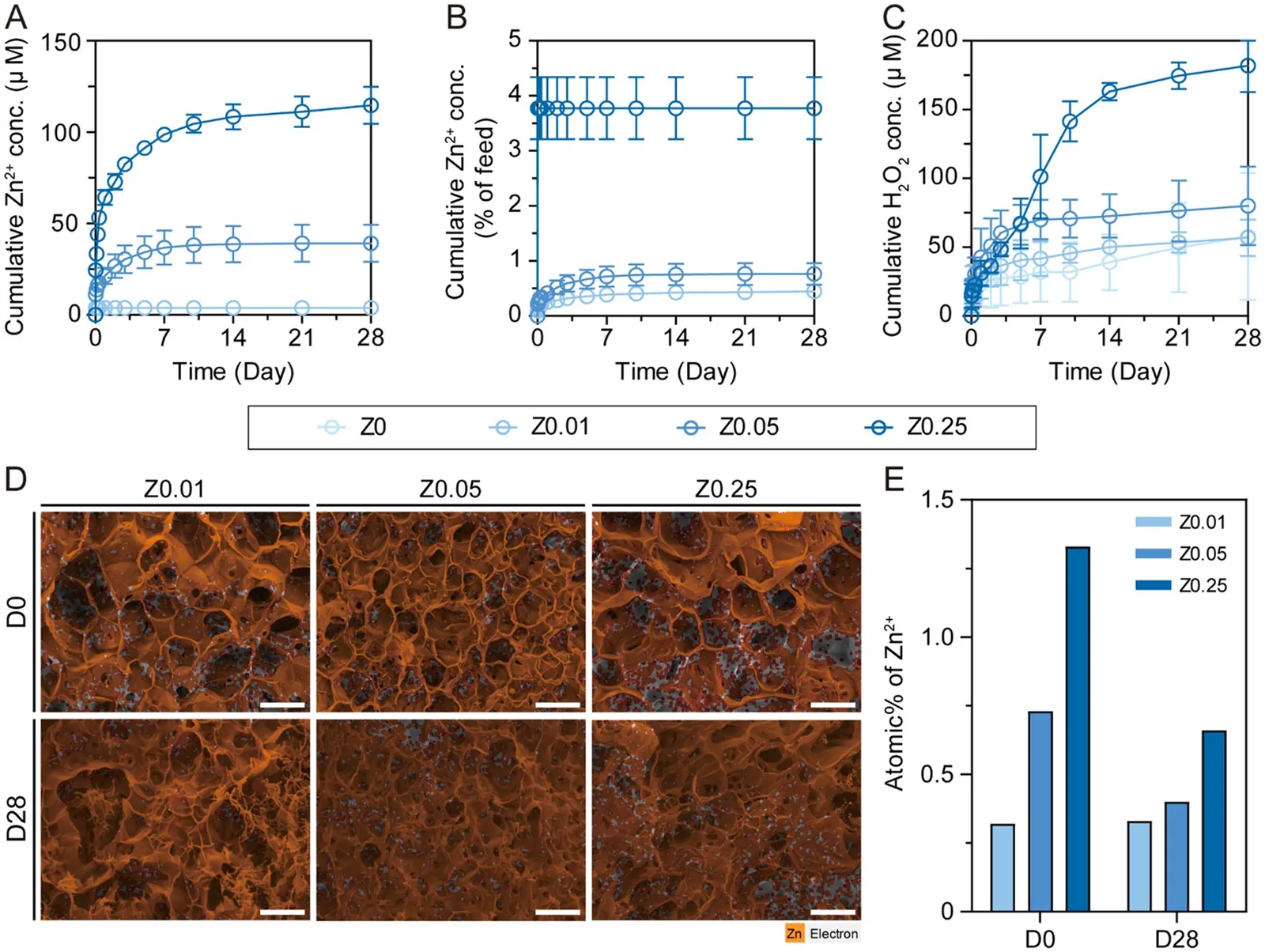
Zn^2+^ and H_2_O_2_ release kinetics from the Zn^2+^-releasing hydrogels. Cumulative Zn^2+^ release as a function of ZnO_2_ concentration, presented as (**A**) released Zn^2+^ concentration and (**B**) release normalized to the initial zinc content (%). (**C**) H_2_O_2_ release depending on ZnO_2_ concentrations. (**D**) SEM-EDS images and elemental mapping of Zn^2+^ retained within the hydrogel matrices at day 0 and day 28. Elemental mapping indicates the distribution of Zn^2+^ within the hydrogel matrices. Scale bars, 100 μm. (**E**) Atomic percentage of Zn^2+^ on day 0 and day 28. Data in (**A–C**) are presented as mean ± SD (*n* = 3). Data in (**E**) are from a single measurement (*n* = 1).

In parallel with Zn^2+^ release, H_2_O_2_ generation increased proportionally with the initial ZnO_2_ concentration (Z0, 23.5 μM; Z0.01, 26.6 μM; Z0.05, 42.6 μM; Z0.25, 31.1 μM for 24 h, Z0, 57.8 μM; Z0.01, 56.9 μM; Z0.05, 80.0 μM; Z0.25, 182.0 μM for day 28) (Figure 2C). The H_2_O_2_ detected in the Z0 group was attributed to background oxidation of residual thiol groups within the GtnSH network during disulfide bond formation [65]. During the first 24 h, cumulative H_2_O_2_ levels in all groups remained below 200 μM, a concentration reported to induce oxidative stress and cellular damage in mammalian cells [64, 66]. These results suggest that the early H_2_O_2_ levels observed in our system are not expected to inhibit cell proliferation under the present experimental conditions. At the same time, given that low levels of H_2_O_2_ may contribute to tissue regeneration, local H_2_O_2_ concentrations should be carefully considered when designing H_2_O_2_-releasing hydrogels.

To assess long-term Zn^2+^ retention, semi-quantitative SEM-EDS analysis was performed. Surface elemental mapping confirmed that Zn^2+^ remained distributed within the matrix even after 28 days of *in vitro* incubation (Figure 2D). In addition, Zn^2+^ was detectable on day 28, with the atomic percentage showing a gradual decline over time (Figure 2E). These findings suggest prolonged retention of Zn^2+^ within the hydrogel matrix, consistent with the sustained release profile. However, these SEM-EDS results should be interpreted as representative, semi-quantitative evidence rather than definitive quantitative data.

To further determine whether the retained Zn^2+^ was physically entrapped or chemically associated with the hydrogel network, high-resolution XPS analyses of the S 2p and Zn 2p regions were performed. Compared with the Z0 group, the Z0.25 group exhibited a newly observed low-binding-energy S 2p doublet at 161.26/162.36 eV, consistent with thiol-Zn^2+^ coordinated sulfur (Supplementary Figure S2A and B). In the Z0 group, the Zn 2p signal was not observed, as expected. In contrast, the Zn 2p spectrum of the Z0.25 group was deconvoluted into two distinct Zn 2p doublets at 1021.39/1044.47 eV and 1022.18/1045.28 eV, indicating the coexistence of at least two Zn coordination environments within the hydrogel matrix (Supplementary Figure S2C and D) [67, 68]. Thus, the XPS results support the presence of partial thiol-Zn^2+^ coordination in the hydrogel matrix, suggesting that prolonged Zn retention is associated not only with physical confinement but also with local chemical interactions that retard Zn^2+^ release.

This observation confirms long-term Zn^2+^ retention within the hydrogel matrix. Such retention arises from thiol-Zn^2+^ coordination, which may delay ion dissociation. The retained Zn^2+^ is expected to be released progressively through diffusion and hydrogel degradation *in vivo* [33]. Although the *in vivo* release behavior and a quantitative degradation-release correlation were not directly established in the present study, these findings support sustained local Zn^2+^ availability rather than rapid loss from the matrix. Taken together, these findings demonstrate that Zn^2+^-releasing hydrogels provide sustained Zn^2+^ release and support a local Zn^2+^ microenvironment.

### Cyto- and tissue-compatible Zn^2+^-releasing hydrogels

Prior to *in vivo* applications, it is essential to confirm that the scaffolds induce neither cellular toxicity nor systemic tissue toxicity upon implantation. Given that our target application is skin tissue regeneration, we utilized HDFs, the predominant cell type in the dermis, to evaluate cytocompatibility [69, 70]. Furthermore, as degradation products of implanted hydrogels can potentially induce systemic toxicity, systemic tissue compatibility was also assessed *in vivo*.

To evaluate cytocompatibility, HDFs were seeded onto well plates and then incubated with the Zn^2+^-releasing hydrogels in culture medium for 24 h. Bright-field and live/dead staining images demonstrated that cells across all groups remained viable and maintained a well-spread morphology (Figure 3A). The WST-1 assay results indicated that all hydrogel-treated groups had higher cell viability than the TCPS control (TCPS, 100%; Z0, 117%; Z0.01, 113%; Z0.05, 110%; Z0.25, 122%) (Figure 3B). These results indicate the excellent cytocompatibility of the Zn^2+^-releasing hydrogels. Together with the H_2_O_2_ release data, these findings suggest that the transient oxidative environment and Zn^2+^ exposure associated with ZnO_2_ decomposition did not adversely affect fibroblast viability in our hydrogel system *in vitro*.

**Figure 3:**
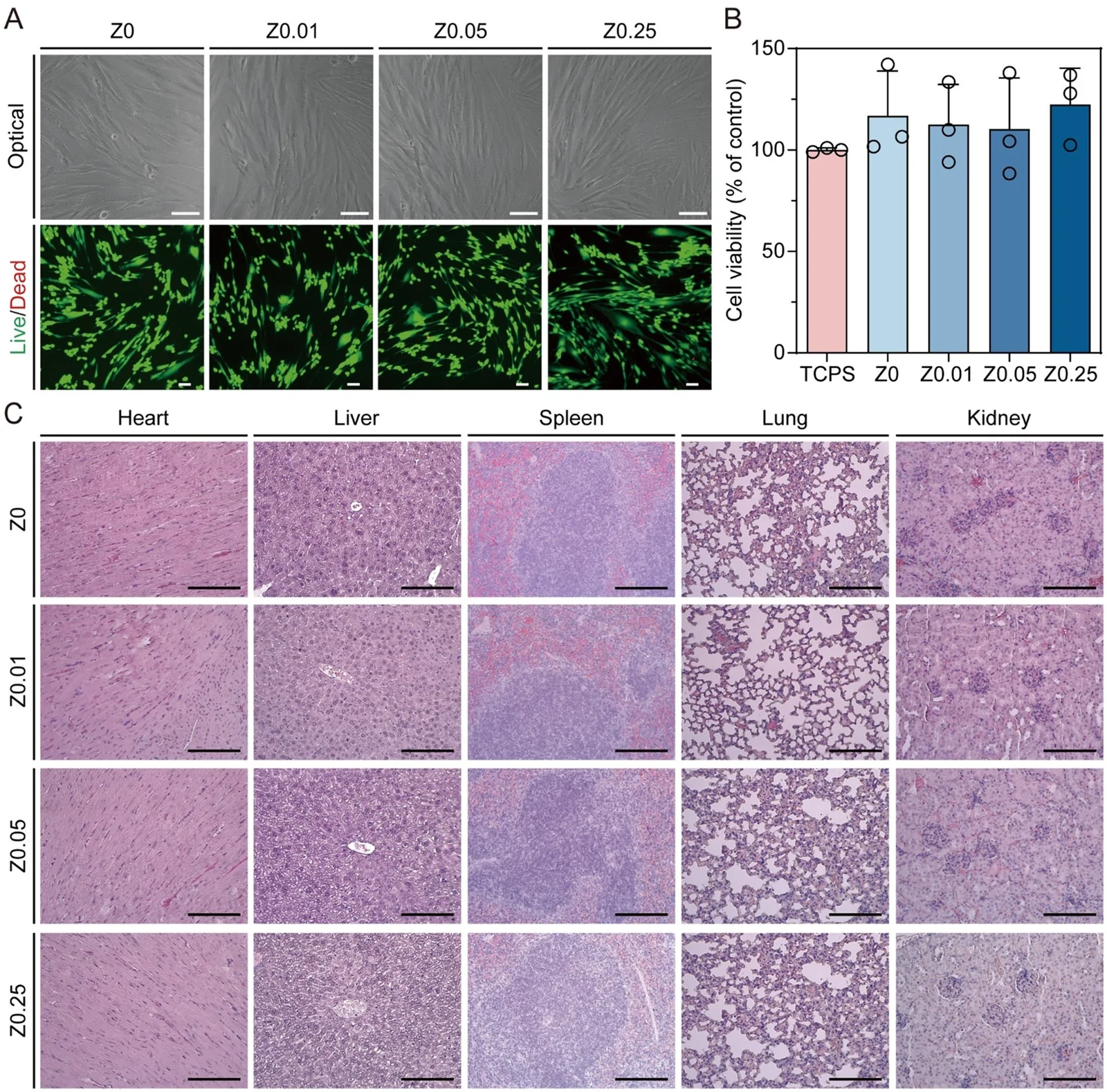
*In vitro* cytotoxicity and *in vivo* histological evaluation of the Zn^2+^-releasing hydrogels. (**A**) Optical and fluorescence micrographs of HDFs cultured with hydrogels for 1 day following live/dead staining. (**B**) Viability of HDFs relative to TCPS at day 1. (**C**) Representative H&E staining images of major organs after complete hydrogel degradation. Scale bars, 100 μm. Data in (**B**) are presented as mean ± SD (*n* = 3), and statistical significance was determined by one-way ANOVA with Tukey’s multiple comparisons test.

Building on these *in vitro* findings, we evaluated systemic tissue compatibility *in vivo*. Four types of hydrogels (Z0, Z0.01, Z0.05, and Z0.25) were implanted subcutaneously in mice. After complete hydrogel degradation, major organs were harvested for histological examination by H&E staining. Systemic toxicity is commonly characterized by inflammatory cell infiltration and tissue damage, including apoptosis or necrosis. However, our H&E results revealed that all major organs maintained normal tissue structure and morphology, with no pathological lesions or toxic damage (Figure 3C). These results indicate that the hydrogels exhibit excellent tissue compatibility.

Overall, the cytocompatibility assays and histological evaluations indicate that the Zn^2+^-releasing hydrogels induce neither cytotoxicity nor systemic tissue damage. While no significant histological changes were observed, the potential for long-term systemic Zn^2+^ accumulation requires further investigation.

### Immunomodulatory properties of the Zn^2+^-releasing hydrogels

Inflammation is a critical phase of wound healing that strongly influences whether tissue regeneration proceeds or chronic pathology develops. In particular, macrophages play a central role during this phase and can adopt either an M1 or M2 phenotype [71]. M1 macrophages are generally associated with inflammatory defense, whereas M2 macrophages support resolution of inflammation and tissue remodeling [72]. Therefore, effective wound healing requires the recruitment of macrophages together with their shift toward an M2-like phenotype [48]. Given that Zn^2+^ can modulate immune responses [73], we assessed the immunomodulatory effects of the Zn^2+^-releasing hydrogels *in vivo* by quantifying macrophage infiltration and phenotypic transition.

To evaluate these properties, the hydrogel explants were excised on day 14. Following histological processing, the infiltrated cells were analyzed by F4/80 (Figure 4A) and F4/80/CD206 double IF staining (Figure 4C). Quantitative assessment was performed to determine the percentage of F4/80^+^ cells in the hydrogel matrix. The proportion of F4/80^+^ cells was higher in the Z0.05 and Z0.25 groups than in the Z0 group (Z0, 13%; Z0.01, 15%; Z0.05, 26%; Z0.25, 24%) (Figure 4B). Furthermore, the proportion of CD206^+^ F4/80^+^ cells among total F4/80^+^ cells was higher in the Z0.25 group than in the Z0 group (Z0, 7%; Z0.01, 26%; Z0.05, 9%; Z0.25, 35%) (Figure 4D), suggesting that Zn^2+^ release may contribute to the formation of a pro-regenerative immune microenvironment.

**Figure 4:**
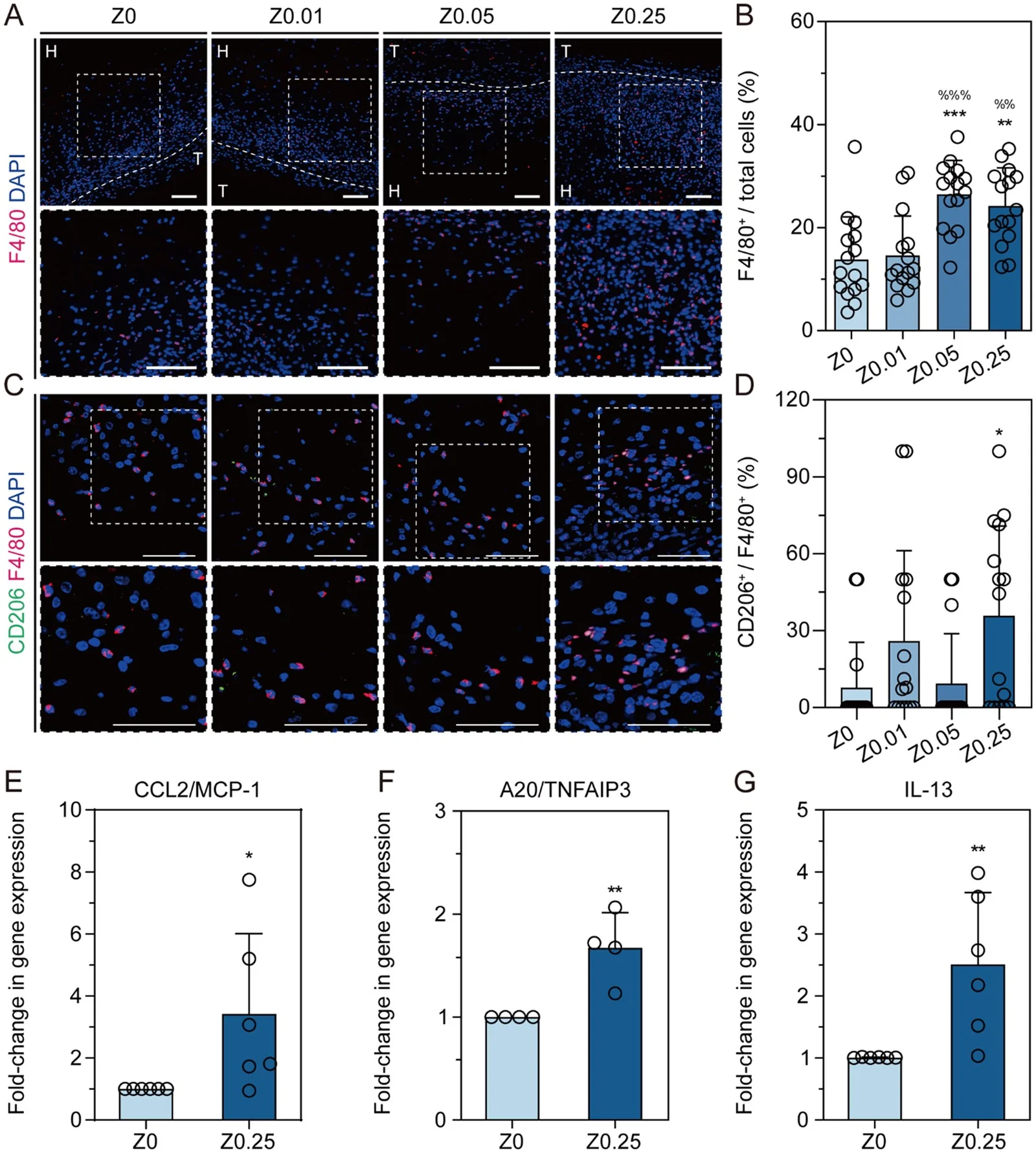
*In vivo* macrophage recruitment and immunomodulatory effects of the Zn^2+^-releasing hydrogels. (**A**) F4/80 IF staining on day 14 (hydrogel: H; surrounding tissue: T). Scale bars, 100 μm. (**B**) Quantification of F4/80^+^/total cells (%). (**C**) CD206/F4/80 double IF staining on day 14 (hydrogel: H; surrounding tissue: T). Scale bars, 50 μm. (**D**) Quantification of CD206^+^/F4/80^+^ (%). Gene expression analysis of infiltrated cells within the hydrogels on day 14 for (**E**) CCL2/MCP-1, (**F**) A20/TNFAIP3, and (**G**) IL-13. Data in (**B, D**) are presented as mean ± SD (*n* = 3; five random microscopic fields per sample), where *n* denotes independent animals. Statistical significance was determined by one-way ANOVA with Tukey’s multiple comparisons test. Data in (**E, G**) are presented as mean ± SD (*n* = 6) and data in (**F**) are presented as mean ± SD (*n* = 4), where *n* denotes independent hydrogel samples implanted subcutaneously in mice. Statistical significance was determined by two-tailed unpaired *t*-tests. * and % indicate significant differences compared to the Z0 and Z0.01 groups, respectively (*P < 0.05, **P < 0.01, ***P < 0.001, ^%%^*P *< 0.01, and ^%%%^*P *< 0.001).

Based on these IF results, we sought to identify key factors driving immune modulation. We hypothesized that released Zn^2+^ modulates the immune response by upregulating cytokines and signaling factors involved in cell–cell communication. To investigate this, hydrogel explants were harvested on day 14 post-implantation, and infiltrated cells were isolated by collagenase digestion. The expression of immunomodulatory genes, including CCL2/MCP-1 (Figure 4E), A20/TNFAIP3 (Figure 4F), and IL-13 (Figure 4G), was analyzed using qRT-PCR. Our results demonstrated that the Z0.25 group exhibited pronounced upregulation of these genes (CCL2/MCP-1, 3.4-fold; A20/TNFAIP3, 1.7-fold; IL-13, 2.5-fold). Functionally, CCL2/MCP-1 is a key chemokine that promotes monocyte and macrophage recruitment [35, 39, 74], whereas A20/TNFAIP3 acts as an anti-inflammatory regulator that attenuates NF-κB signaling [40, 41]. In addition, IL-13 facilitates alternative macrophage activation and M2 polarization [42, 75–77]. These results suggest that Zn^2+^ release enhances the expression of immune-regulatory mediators compared with the Zn^2+^-free group.

Elevated CCL2/MCP-1 is consistent with enhanced recruitment of macrophages into the hydrogel, aligning with the increased number of F4/80^+^ cells. In parallel, increased A20/TNFAIP3 suggests reinforcement of anti-inflammatory feedback regulation. Moreover, the upregulation of IL-13 supports alternative macrophage activation, consistent with the increased number of CD206^+^ cells. Overall, these data indicate that the Zn^2+^-releasing hydrogels enhance macrophage recruitment and promote macrophage phenotypic transition, which may help create an immune environment supportive of tissue regeneration.

### Enhanced cell proliferation and angiogenesis

After the inflammatory phase, wound healing enters the proliferative phase, which is crucial for tissue restoration [78, 79]. This stage is marked by increased cell migration and proliferation [80]. Efficient progression from inflammation to proliferation is essential for proper tissue repair, and M2 macrophages contribute to this transition through the release of pro-regenerative cytokines and growth factors [81]. Based on our observation that the Zn^2+^-releasing hydrogels enhance M2 polarization, we next examined whether this immunomodulation translated into improved cell proliferation and neovascularization *in vivo*.

To assess proliferation and angiogenesis, IF staining was performed on hydrogel explant sections. We utilized Ki67 to detect proliferative cells (Figure 5A) and CD31 to visualize vascular endothelial cells (Figure 5C). The Zn^2+^-releasing hydrogel groups exhibited a greater proportion of Ki67^+^ cells than the Z0 group (Z0, 23%; Z0.01, 27%; Z0.05, 30%; Z0.25, 31%) (Figure 5B). In addition, the percentage of CD31^+^ cells among total cells increased with increasing ZnO_2_ content (Z0, 21%; Z0.01, 25%; Z0.05, 29%; Z0.25, 32%) (Figure 5D).

**Figure 5:**
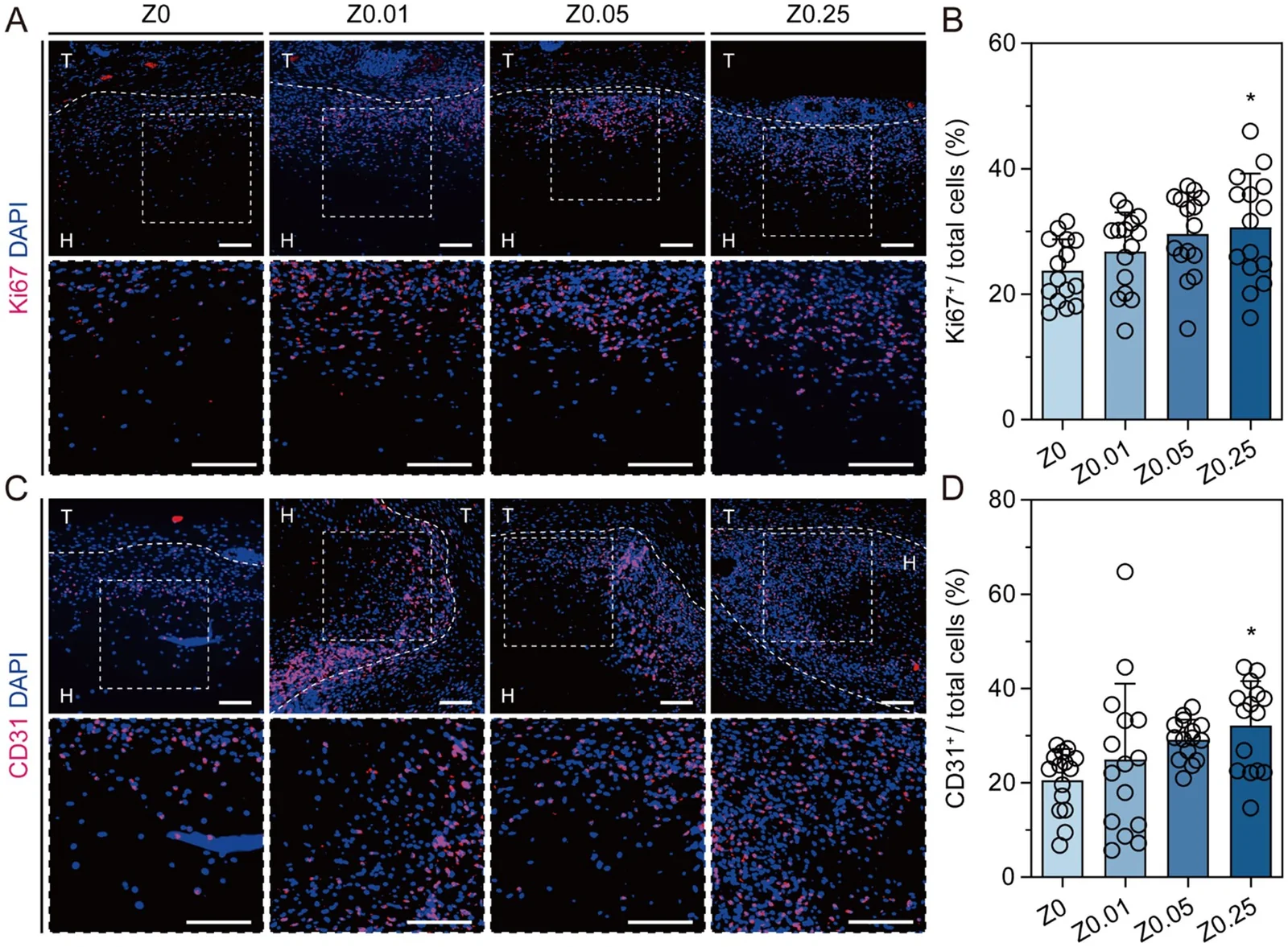
*In vivo* proliferative and angiogenic effects of the Zn^2+^-releasing hydrogels. (**A**) Ki67 IF images on day 14 (hydrogel: H; surrounding tissue: T). Scale bars, 100 μm. (**B**) Quantification analysis of Ki67^+^/total cells (%). (**C**) CD31 IF images on day 14 (hydrogel: H; surrounding tissue: T). Scale bars, 100 μm. (**D**) Quantification of CD31^+^/total cells (%). Data in (**B, D**) are presented as mean ± SD (*n* = 3; five random microscopic fields per sample), where *n* denotes independent animals. Statistical significance was determined by one-way ANOVA with Tukey’s multiple comparisons test. * indicates a significant difference compared to the Z0 group (*P < 0.05).

The enhanced proliferative and angiogenic responses may reflect the combined contributions of the hydrogel-modulated immune microenvironment and the effects of Zn^2+^. It has been reported that M2 macrophages secrete paracrine mediators, such as transforming growth factor-β (TGF-β), platelet-derived growth factor (PDGF), and vascular endothelial growth factor [82, 83], which can support cell proliferation and angiogenesis. In addition, previous studies have shown that Zn^2+^ contributes to wound healing by promoting proliferative and angiogenic responses. Although we did not directly quantify these growth factors at the protein level or isolate the direct effects of Zn^2+^, the increased proportions of Ki67^+^ and CD31^+^ cells indicate a local environment favorable for proliferation and neovascularization. Taken together, these findings suggest that the Zn^2+^-releasing hydrogels promote proliferative and angiogenic responses *in vivo*.

### Improved wound healing and tissue remodeling

Zn^2+^ exhibits therapeutic effects by influencing multiple wound-healing processes, such as immune modulation, cellular proliferation, angiogenesis, and tissue remodeling [84, 85]. Building on our previous findings that the Zn^2+^-releasing hydrogels promoted macrophage polarization, cell proliferation, and angiogenesis, we hypothesized that these combined effects would promote faster wound closure and enhance tissue regeneration.

To confirm the therapeutic efficacy of the Zn^2+^-releasing hydrogels, a critical-size skin defect model was established in mice (Figure 6A). An 8-mm dorsal skin defect was generated, and 50 μL of each hydrogel (Z0, Z0.01, Z0.05, and Z0.25) was applied. Wound closure (%) was monitored over 14 days (Figure 6B). The Z0.25 hydrogel-treated group showed accelerated wound closure. By day 7, the Z0.25 group achieved a closure rate of 68%, whereas the other groups showed slower wound closure rates (Z0, 46%; Z0.01, 44%; Z0.05, 48%) (Figure 6C). These results suggest that the Zn^2+^-releasing hydrogels mainly contribute to accelerated early wound repair and improved tissue regeneration, rather than reducing the final wound area.

**Figure 6:**
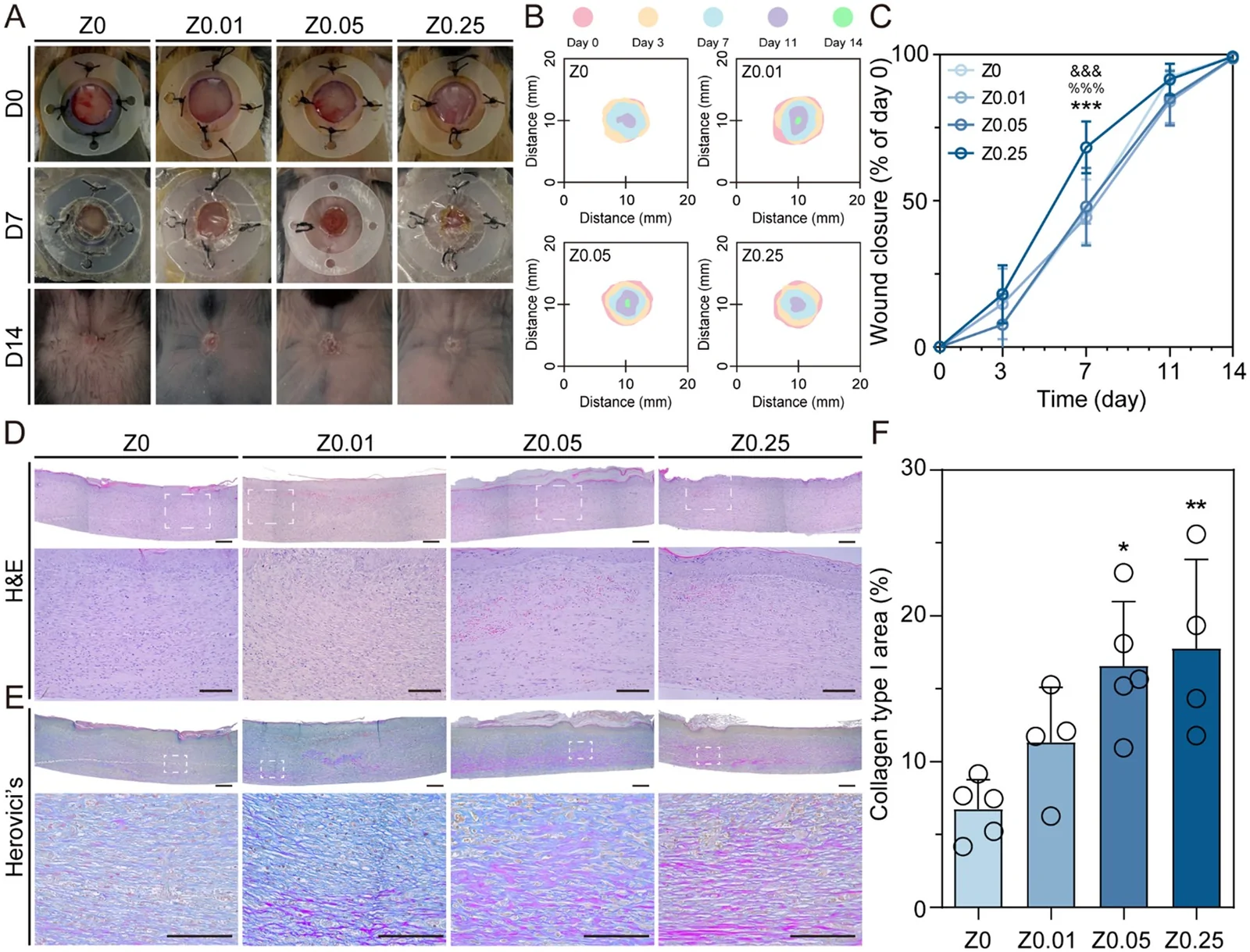
Effects of Zn^2+^-releasing hydrogels on tissue regeneration and maturation in a critical-size skin defect model. (**A**) Representative digital images of wounds in the critical-size skin defect model. (**B**) Schematic diagrams illustrating the reduction in wound area for each group on days 3, 7, 11, and 14. (**C**) Quantitative analysis of wound closure over time. (**D**) H&E staining images of the regenerated wounds on day 14. (**E**) Herovici’s staining to distinguish mature type I collagen from immature type III collagen. Scale bars, 100 μm. (**F**) Quantitative analysis of type I collagen area. Data in (**C, F**) are presented as mean ± SD (*n* = 5 for Z0 and Z0.05, and *n* = 4 for Z0.01 and Z0.25), where *n* denotes independent animals with one wound analyzed per animal. Statistical significance was determined by two-way ANOVA with Tukey’s multiple comparisons test for (**C**) and by one-way ANOVA with Tukey’s multiple comparisons test for (**F**). *, %, and & indicate significant differences compared to the Z0, Z0.01, and Z0.05 groups, respectively (*P < 0.05, **P < 0.01, ***P < 0.001, ^%%%^*P *< 0.001, and ^&&&^*P *< 0.001).

Beyond the rate of closure, the structural resemblance of regenerated tissue is crucial. To evaluate this, H&E staining was performed. The Z0.25 group exhibited a fully re-epithelialized epidermis and a well-organized dermis (Figure 6D). These findings indicate that the Zn^2+^-releasing hydrogels support not only re-epithelialization but also functional tissue regeneration that closely resembles native skin architecture.

Wound remodeling further requires restoration of ECM organization. Collagen maturation, specifically the progression from immature type III collagen to mature type I collagen, is fundamental for re-establishing tissue tensile strength and integrity [86]. To investigate this, Herovici’s staining was used, which differentiates young collagen (blue) from mature collagen (purple) (Figure 6E). The Z0.25 group showed the highest proportion of mature type I collagen deposition (Z0, 7%; Z0.01, 11%; Z0.05, 17%; Z0.25, 18%) (Figure 6F). These results suggest that the Zn^2+^-releasing hydrogels promote collagen maturation, thereby enhancing wound remodeling and structural resemblance to normal skin.

As discussed previously, M2 macrophages have been reported to secrete fibrogenic mediators, including TGF-β and PDGF. These factors can increase myofibroblast activity and upregulate α-smooth muscle actin and type I collagen expression [87, 88]. Thus, our results suggest that controlled Zn^2+^ release coordinates immune modulation with collagen remodeling, ultimately promoting functional skin regeneration.

## Conclusion

In this study, we developed Zn^2+^-releasing immunomodulatory hydrogels that enhanced skin tissue regeneration through controlled Zn^2+^ release. We fabricated a dual-crosslinked network incorporating ZnO_2_ within functionalized gelatin polymers and established that thiol-Zn^2+^ coordination enabled sustained long-term ion release without systemic toxicity. This unique mechanism effectively addressed the limitations of rapid ion dissociation often observed in conventional metal-based therapies. Central to our findings was the ability of the hydrogel to modulate macrophage phenotypes. The sustained release of Zn^2+^ upregulated immunomodulatory and regenerative genes (CCL2/MCP-1, A20/TNFAIP3, and IL-13) and biased the macrophage population toward the M2 phenotype. Additionally, the Zn^2+^-releasing hydrogels exhibited potent regenerative capacity *in vivo*, promoting immune modulation, cell proliferation, angiogenesis, and the deposition of mature type I collagen. These biological responses facilitated the functional recovery of native skin architecture. These findings suggest that our Zn^2+^-releasing hydrogels are a promising alternative to passive wound-healing materials. Nevertheless, several limitations should be acknowledged. A quantitative degradation-release correlation was not established in the present study, and further investigation is required to clarify how hydrogel degradation influences Zn^2+^ release kinetics *in vivo*. In addition, potential risks associated with long-term systemic Zn^2+^ accumulation should be further evaluated, particularly in the liver and kidneys, which play central roles in Zn^2+^ clearance.

## Supplementary Material

rbag147_Supplementary_Data
