# Supplementary material for: Zinc ion-releasing immunomodulatory hydrogels for enhanced wound healing
Source: Regen Biomater. 2026 Jul 13;13:rbag147. doi: 10.1093/rb/rbag147 (PMC13629550; doi:10.1093/rb/rbag147)
Supplement: Supplementary file 1 [file rbag147_supplementary_data.docx]

**Supplementary Data**

**Zinc ion-releasing immunomodulatory hydrogels for enhanced wound healing**

Yeonjeong Kim^a,†^, Minjae Heo^a,†^, Jeong Min Kim^a^, Ki Dong Park^b,*^, Kyung Min Park^a,c,*^

^a^Department of Bioengineering and Nano-Bioengineering, Incheon National University, Incheon, 22012, Republic of Korea

^b^Department of Molecular Sciences and Technology, Ajou University, Gyeonggi-do, 16499, Republic of Korea

^c^Research Center for Bio Materials & Process Development, Incheon National University, Incheon, 22012, Republic of Korea

†These authors contributed equally to this work.

Supplementary Figure S1. *In vivo* cellular infiltration into the Zn^2+^-releasing hydrogels. (A) Schematic representations of *in vivo* subcutaneous implantation. H&E-stained images of implanted hydrogels depending on (B) polymer concentration and thiol equivalence, and (C) ZnO_2_ concentration. Scale bars, 100 µm. Quantitative results of (D) polymer concentration, (E) thiol equivalence, and (F) ZnO_2_ concentration. (D, F) are presented as mean ± s.d. (n=3; five random microscopic fields per sample). Statistical significance was determined by one-way ANOVA with Tukey’s multiple comparisons test. (E) is presented as mean ± s.d. (n=3; five random microscopic fields per sample). Statistical significance was determined by a two-tailed unpaired t-test. *, ^%^, and ^&^ indicate differences compared to the Z0, Z0.01, and Z0.05 groups, respectively (***p* < 0.01, *****p* < 0.0001, ^%%^*p* < 0.01, and ^&&^*p* < 0.01).

Supplementary Figure S2. High-resolution XPS spectra analysis of Z0 and Z0.25 groups. S 2p region for the surfaces of the (A) Z0 and (B) Z0.25 hydrogels, and Zn 2p region for the surfaces of the (C) Z0 and (D) Z0.25 hydrogels. Colored peaks indicate deconvoluted components assigned to different chemical states.
